# Parasite load and genotype are associated with clinical outcome of piroplasm-infected equines in Israel

**DOI:** 10.1186/s13071-020-04133-y

**Published:** 2020-05-20

**Authors:** Sharon Tirosh-Levy, Amir Steinman, Hadas Levy, Yotam Katz, Margarita Shtilman, Yuval Gottlieb

**Affiliations:** Koret School of Veterinary Medicine, The Robert H. Smith Faculty of Agriculture, Food and Environment, The Hebrew University of Jerusalem, Rehovot, Israel

**Keywords:** *Theileria equi*, *Babesia caballi*, Equine piroplasmosis, Phylogeny, Parasitemia, Clinical signs

## Abstract

**Background:**

Equine piroplasmosis is a highly endemic protozoan disease of horses worldwide, caused by *Theileria equi* and *Babesia caballi*. While most horses in endemic areas are subclinically infected, the mechanisms leading to clinical outcome are vastly unknown. Moreover, since clinical signs of disease are not specific, and the prevalence in endemic areas is high, it is difficult to determine if equine piroplasmosis is the cause of disease. To identify possible mechanisms leading to the clinical outcome in an endemic area, we compared parasite loads and genotypes in clinically and subclinically infected horses.

**Methods:**

Blood was collected from horses with clinical signs consistent with equine piroplasmosis, and from apparently healthy horses in Israel. Packed cell volume and total solids were measured. Quantitative and diagnostic polymerase chain reaction were used to identify, quantify and classify equine piroplasmosis infection. Phylogenetic analyses were used to determine the genotype of both parasites.

**Results:**

For both parasites, clinical cases were associated with low mean packed cell volume and high mean parasite load (*P* < 0.001), enabling the determination of a cut-off value to distinguish between clinically and subclinically infected horses. Samples of *Theileria equi* from subclinical horses were classified into three different *18S* rRNA genotypes, D (*n* = 23), A (*n* = 12) and C (*n* = 5), while samples from all clinical cases (*n* = 6) were classified as genotype A. The sequences of *T. equi* equi merozoite antigens 1 (*ema-1*, *n* = 9) and 2 (*ema-2*, *n* = 11) genes were fairly conserved and did not differ between clinical and subclinical cases*. Babesia caballi* rhoptry associated protein-1 (*rap-1*) was classified into sub-genotypes A1 (*n* = 14) and A2 (*n* = 5) with no association to clinical outcome. Classification of the *18S* rRNA gene (sub-genotypes B1 and B2) agreed with the *rap-1* classification.

**Conclusions:**

The results of this study suggest that quantification of parasite loads of infected horses may be used to distinguish between infections resulting in disease and subclinical cases. Although number of clinical cases is limited, we identified *T. equi 18S* rRNA genotype A to be associated with clinical disease. This finding emphasizes the importance of in-depth genetic characterization of *T. equi* genotypes to identify possible markers for virulence.
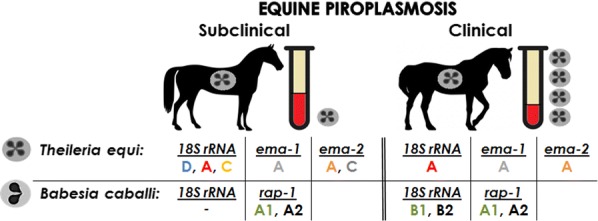

## Background

Equine piroplasmosis (EP) is an important tick-borne disease of equids, caused by the hemoprotozoan apicomplexan parasites *Theileria equi* and *Babesia caballi.* Both parasites are endemic in most parts of the world: South America, Africa and most parts of Asia and Europe, including Israel [1–3]. Introduction of these parasites to non-endemic areas, in which suitable tick vectors are prevalent, may cause epizootic spread of disease [1, 4]. *Theileria equi* is usually more prevalent than *B. caballi* in endemic regions, has more severe clinical manifestation and may lead to life-long infestation if untreated [1, 2]. Economic consequences of infection include medical treatment and mortality of clinically infected animals, reduced performance of infected animals and restrictions on animal transport between endemic and non-endemic areas [1, 2].

Clinical disease is usually characterized by acute hemolytic anemia, and ranges from subclinical, non-apparent parasite carriage to peracute, life-threatening disease. Clinical signs of acute disease include anemia, jaundice, inappetence, edema and pigmenturia. Most infected horses become asymptomatic carriers after resolution of clinical signs [1, 2]. The factors contributing to the development of clinical disease are unclear. Host innate immunity plays a central role in the immune response, while adaptive immunity is also essential as high antibody titer correlates with parasite control [1, 5]. Early exposure in endemic areas usually leads to protective immunity, while primary exposure of naïve adults more often leads to clinical disease. However, clinical cases in adult horses are also reported in endemic areas [1, 2].

Apicomplexan parasites, including *T. equi* and *B. caballi,* show genetic diversity [6–9]. Five *T. equi* and two *B. caballi 18S* rRNA genotypes have been described [3, 10], and none was associated with parasite virulence. In recent years, *18S* rRNA based classification has been questioned, as evidences for considerable genetic variation in other loci led to the description of several potential new species [7, 8]. The novel species *Theileria haneyi* has *18S* rRNA sequence similar to genotype C but has a much smaller and considerably different genome compared to *T. equi* [8], and three variants of *Theileria africa* have *18S* rRNA sequences similar to genotype D, but differs from *T. equi* in its *30S* rRNA gene sequence [7].

In order to detect genetic variation that is linked to parasite evasion of the host immune system, known immunogenic proteins have been characterized: in *B. caballi*, rhoptry associated protein-1 (*rap-1*) gene and protein [11–13] and in *T. equi,* equi merozoite antigen (*ema*) gene family, which includes nine genes [3, 14–16]. Two *B. caballi rap-1* genotypes have been characterized with considerable differences in protein structure [11], jeopardizing the results of the United States Department of Agriculture (USDA)-approved cELISA detection kit (based on the RAP-1 protein) on isolates from Africa and the Middle East [11–13]. The *T. equi ema-1* and *ema-2* genes and proteins are more conserved, and have been used for the development of serological assays [17, 18]. Some sequence variation has been detected for these genes, although little diversity is found among isolates within a geographical region [3, 14, 19].

The aim of this study was to identify possible associations between parasite genotype and parasite density and the clinical outcome in *T. equi* and *B. caballi* infection in an endemic area, by comparing clinical with subclinical cases in Israel. The use of quantitative tools and additional parasite gene sequences were applied to overcome the limitations of classification based solely on the *18S* rRNA gene.

## Methods

### Sample collection

Veterinarians listed in the Israeli Association of Equine Practitioners were asked to send blood samples from any case suspected as EP. The clinical evaluation was left to the attending veterinarian’s discretion. Most referred cases exhibited one or more of the following clinical signs: fever, anemia, icterus, inappetence and documented or suspected exposure to ticks. Blood samples (*n* = 25) were diagnosed molecularly as described bellow, and tested for packed cell volume (PCV), when available. Only horses with confirmed infection to either of the parasites by PCR and sequencing, were included in the study (six *T. equi* and six *B. caballi*).

Blood samples from apparently healthy horses were collected as a part of a surveillance study designed to represent the distribution of the horse population in Israel (*n* = 395). Blood from all horses was collected from the jugular vein into sterile vacuum tubes containing ethylenediamine tetraacetic acid (EDTA). Packed cell volume and total solids (TS) were measured using standard methodology prior to storage of whole blood at -20 °C until processing. All blood samples were screened for the presence of both parasites using PCR. For *B. caballi*, all samples which were PCR-positive and successfully sequenced, were included in the analysis (*n* = 13). For *T. equi*, five samples were selected from each of surveillance farms showing more than five positive horses and were used for the molecular characterization. Forty samples with good quality sequences were ultimately used in the analysis. The characteristics of all horses included in this study are listed in Additional file 1: Table S1.

### Identification and characterization of EP parasites using polymerase chain reaction (PCR)

DNA was extracted as described in Tirosh Levy et al. [20], and initial screening for infestation with EP parasites was performed using diagnostic PCR directed to identify a 400-bp fragment of *T. equi 18S* rRNA gene [21], and *B. caballi rap-1* gene [11], as previously described [3, 12, 22] (for primer list, see Additional file 2: Table S2).

The molecular characterization of *T. equi* was based on amplification and sequencing of its *18S* rRNA, *ema-1* and *ema-2* genes, while the molecular characterization of *B. caballi* was based on the amplification and sequencing of its *18S* rRNA and *rap-1* genes. The full length (1600 bp) of *T. equi* and *B. caballi 18S* rRNA gene was amplified using primers NBabesia1F and 18SRev-TB primers [6, 23, 24] (Additional file 2: Table S2), as previously described [10]. Full-length sequences were amplified from all available clinical samples and *B. caballi-*positive samples and from 50 *T. equi-*positive subclinical samples, five horses per positive farm. A 750-bp fragment of *T. equi ema-*1 gene was amplified using primers EMA-1F/R [21] (Additional file 2: Table S2). A 800-bp fragment of *T. equi ema-2* gene was amplified using primers EMA-2F/R (Additional file 2: Table S2). A fragment of approximately 1500-bp fragment of *B. caballi rap-1* gene was amplified using the primers Bc9_RAPF/R, as previously described [11, 12] (Additional file 2: Table S2).

All positive PCR products were cleaned using Exonuclease I and Shrimp alkaline phosphatase (New England Biolabs Inc., Massachusetts, USA) and sent for sequencing (Macrogen Europe, Amsterdam, The Netherlands). Sequencing of the complete *18S* rRNA gene was performed using three different sets of primers, as previously described [3, 6] (Additional file 2: Table S2). Sequencing of *ema-1* and *ema-2* genes was performed using both the forward and reverse primers. Sequencing of *rap-1* gene was performed using both the forward and reverse primers as well as the internal set of primers Bc9_RAP2F/R (Additional file 2: Table S2), as previously described [11, 12].

Consensus sequences of each sample and gene were constructed using the Chromas (version 2.6, Technelysium Pty Ltd., South Brisbane, Australia) and MEGA v.7.0.18 software [25] (http://www.megasoftware.net, last accessed November 2018), as previously described [20]. BLAST (http://www.ncbi.nlm.nih.gov/BLAST, last accessed November 2019) analysis confirmed that all constructed sequences were 99–100% identical to previously published sequences of the corresponding gene available on GenBank. The constructed sequences from all samples and genes were submitted to the GenBank database.

Phylogenetic trees were constructed using both Maximum Likelihood and Neighbor-Joining methods in MEGA7, as previously described [20]. The statistical model used for each analysis was selected by the lowest Bayesian Information Criterion (BIC) score, calculated in MEGA7. All algorithms were constructed with bootstrap replicates from 1000 randomly selected samples to estimate reliability.

The divergence between *T. equi ema-2* sequences from this study and from GenBank was estimated using distance matrix analysis, Kimura 2-parameter+G model in MEGA7.

### Quantification of EP parasitemia using quantitative real-time PCR reaction (qPCR)

Quantification of parasitemia was assessed via qPCR using TaqMan® minor groove binder (MGB) (Thermo Fisher Scientific, Waltham, MA, USA) probes targeting *T. equi ema-1* gene [14] and *B. caballi 18S* rRNA gene [26] (Additional file 2: Table S2). A clean PCR product of each gene was used to prepare the standard curve, and gene copy number (gcn) was calculated from the molecular weight and gene length [gcn = (ng × gcn/mole)/(bp × ng/g × g/mole of bp)]. A standard curve of 1–10^8^ copies was used to determine copy number in each sample. The standard curve of each parasite was later compared with DNA extracted from blood with a known percentage of parasitized erythrocytes (%PE, obtained from culture diluted in non-infected horse blood) to extrapolate from gene copies to infected red blood cells [20]. The cut-off for parasite detection was set as one gene copy, equivalent to 6.3 × 10^−5^ % PE for *T. equi* and 8 × 10^−5^ % PE for *B. caballi*.

Parasite quantities of clinical *versus* subclinical cases and of *T. equi versus B. caballi* subclinical cases were compared using Mann-Whitney nonparametric statistical test. Receiver operating characteristic (ROC) curves were generated to establish cut-off values to differentiate parasitemia between clinical and subclinical cases of each parasite. The statistical analysis was performed in SPSS 22.0® software (IBM, Armonk, NY, USA).

## Results

### Study population

Infection with EP parasites was confirmed in 12 horses exhibiting clinical signs consistent with EP including fever, anemia, icterus and inappetence. In six of these cases parasites were identified in blood smear (others were not tested). In the 12 clinical cases, diagnostic PCR identified six cases of *T. equi* infection and six other cases of *B. caballi* infection. One of the *T. equi* cases was a fatal case of a neonatal filly born to a subclinically infected mare [27]. The ages of clinically infected horses ranged between newborn and 17 years-old. Sex, breed, mean age and mean PCV (PCV was available in ten cases) are listed in Table 1.

**Table 1:** Characteristics of the horses participated in this study

	*Babesia caballi*		*Theileria equi*	
	Clinical	Subclinical	Clinical	Subclinical
*n*	6	13	6	40
Mare	3	2	4	16
Gelding	3	10	2	23
Stallion		1		1
Arabian			1	
Tennessee walking horse	1		1	
Quarter horse	3		4	4
Warmblood	1			
Andalusian		1		
Pony				2
Mixed	1	12		34
Mean age	6.20	6.90	12.17	9.17
Mean PCV	19.75	30.70	19.42	33.82

*Note:* The number (*n*) of clinical and subclinical isolates analyzed in this study, sex and breed distribution, mean age and mean packed cell volume (PCV) are specified for each group

Samples from subclinical horses were collected from apparently healthy horses, including 40 *T. equi* infected horses and 13 horses co-infected with both *T. equi* and *B. caballi*. The ages of the subclinically infected horses ranged between 2–22 years. Sex, breed, mean age and mean PCV (available in 59 cases) are listed in Table 1.

The mean PCV of clinically infected horses was significantly lower than of subclinically infected horses (*P* < 0.001, 95% confidence interval, CI: − 17.41–11.24 for *T. equi* and *P* < 0.001, 95% CI: − 15.84–6.06 for *B. caballi*, Table 1). The geographical distribution of the collection sites is presented in Additional file 3: Figure S1.

### Quantification of EP parasitemia in clinical *versus* subclinical horses

**Fig. 1:**
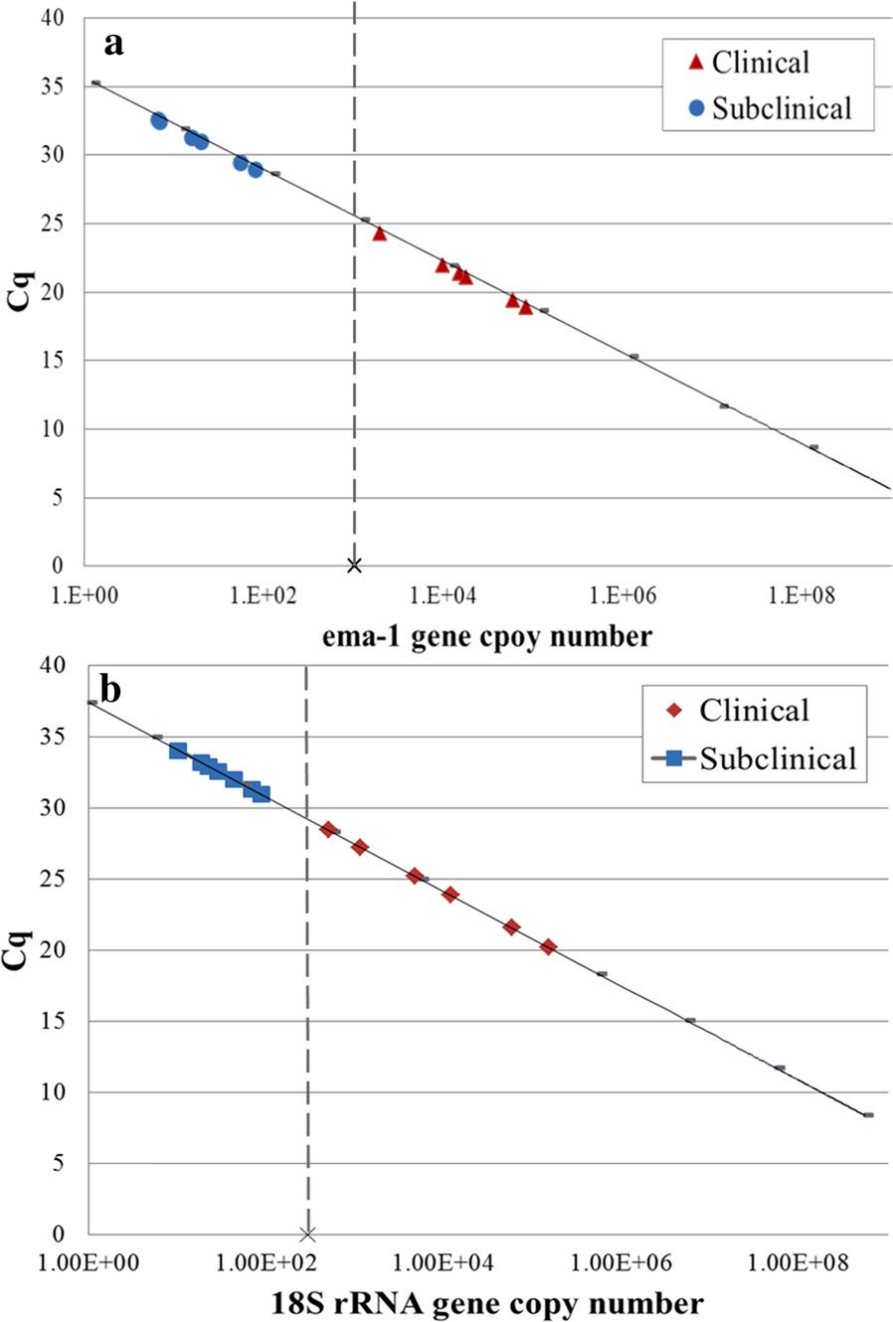
*Theileria equi* (**a**) and *B. caballi* (**b**) parasite loads in clinical (red) and subclinical (blue) horses, as determined by qPCR. For each parasite a standard curve (black line) was created using serial dilutions of a clean PCR product of each gene (gray marks). Parasite gene copy number from field samples was calculated from the quantification cycle (Cq) and the standard curve. A diagnostic cut-off distinguishing between clinical and subclinical cases of each parasite was determined by ROC analysis and the cut-off value is marked by a vertical dashed line

Quantitative real time PCR was used to evaluate *T. equi* parasitemia in five clinically infected and seven, randomly selected, subclinically infected horses. Parasitemia of clinically infected animals ranged between 1998–59,813 *ema-1* gene copies (mean: 21,033.6; SEM: 10,076.2), equivalent to 0.12–3.8% parasitized erythrocytes (PE), while in subclinical animals it ranged between 5–81 gene copies (mean: 27.3; SEM: 11.2), equivalent to 0.0003–0.005% PE (Fig. 1a). The difference between parasite loads of clinical *versus* subclinical animals was statistically significant (Mann-Whitney, *P* = 0.003). A diagnostic cut-off was calculated as 1040 gene copies or 0.066% PE with a sensitivity and specificity of 100% (ROC AUC = 1, 95% CI: 1, *P* = 0.004).

Quantitative real time PCR was used to evaluate *B. caballi* parasitemia in six clinically infected and seven, randomly selected, subclinically infected horses. Parasitemia of clinically infected animals ranged between 503–152,696 *18S* rRNA gene copies (mean: 38,348; SEM: 24,587.7), equivalent to 0.007–2.11% PE, while in subclinical animals it ranged between 10–88 gene copies (mean: 40.5; SEM: 10.9), equivalent to 0.0001–0.0012% PE (Fig. 1b). The difference between parasite loads of clinical *versus* subclinical animals was statistically significant (*P* = 0.001). A diagnostic cut-off was calculated as 296 gene copies or 0.004% PE with a sensitivity and specificity of 100% (ROC AUC = 1, 95% CI: 1, *P* = 0.003).

### Classification of *T. equi* and *B. caballi* based on *18S* rRNA gene

The *18S* rRNA gene was successfully amplified and sequenced in all *T. equi* clinical and subclinical samples (GenBank: MK392050-MK392061, MN611313-MN611352) and from all *B. caballi* clinical samples (GenBank: MK288106-MK288110, MN629354). All *B. caballi-*positive subclinical horses were co-infected with *T. equi* and, therefore, could not have been classified based on their *18S* rRNA gene.

**Fig. 2:**
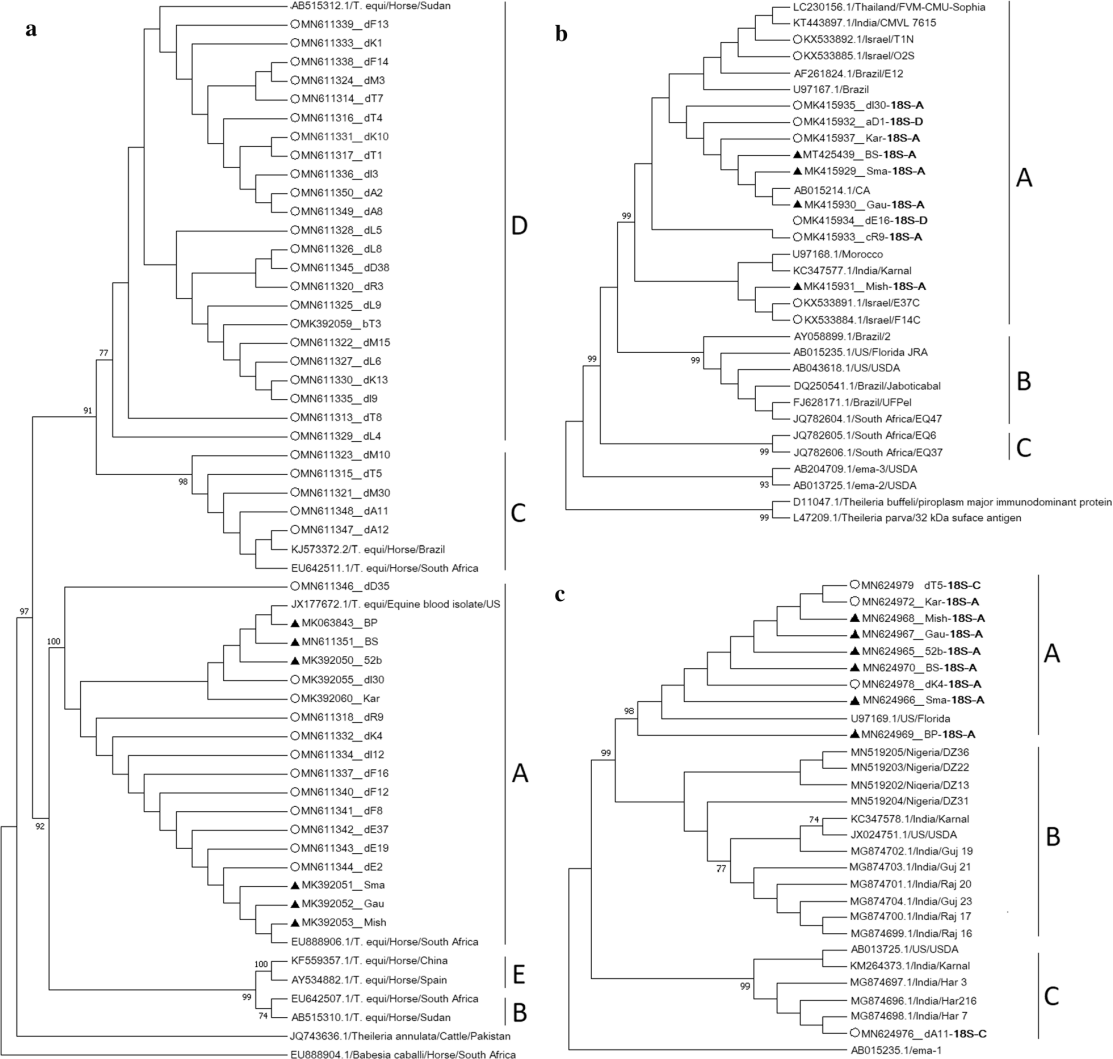
Phylogenetic analysis of *T. equi* sequences obtained from clinically infected horses (triangles) and subclinically infected horses (open circles) using three genes (sample names as detailed in Additional file 1: Table S1). **a** Analysis of 1079 nucleotide positions of *T. equi 18S* rRNA gene, from 6 clinical and 40 subclinical samples, along with additional published sequences (GenBank ID/parasite/host/location). The phylogenetic tree was constructed based on the Tamura-Nei model with gamma distribution (+G). **b** Analysis of 400 nucleotide positions of *T. equi ema-1* gene sequences obtained from four clinical and five subclinical samples, along with additional published sequences (GenBank ID/parasite/host/location). The classification of each sample according to its *18S* rRNA gene is states near the sample name (− 18SX). The phylogenetic tree was constructed based on the Kimura 2-parameter model with consideration on invariable sites (+ I). **c** Analysis of 782 nucleotide positions of *T. equi ema-2* gene sequences obtained from six clinical and four subclinical samples, along with all 19 additional published sequences (GenBank ID/parasite/host/location). The classification of each sample according to its *18S* rRNA gene is states near the sample name (− 18SX). The phylogenetic tree was constructed by based on the Kimura 2-parameter model with consideration on invariable sites (+ I). All phylogenetic trees were constructed using maximum likelihood method and 1000 bootstrap replicates. The percentage of trees in which the associated samples clustered together is shown next to the branches when it was above 70%. The analysis was constructed in MEGA7

Out of the five *T. equi 18S* rRNA genotypes (A–E) [6, 28], all six clinical samples and 12 (30%) subclinical samples were classified as genotype A; 23 (57.5%) subclinical isolates were classified as genotype D and the remaining five (12.5%) subclinical isolates were classified as genotype C (Fig. 2a).

**Fig. 3:**
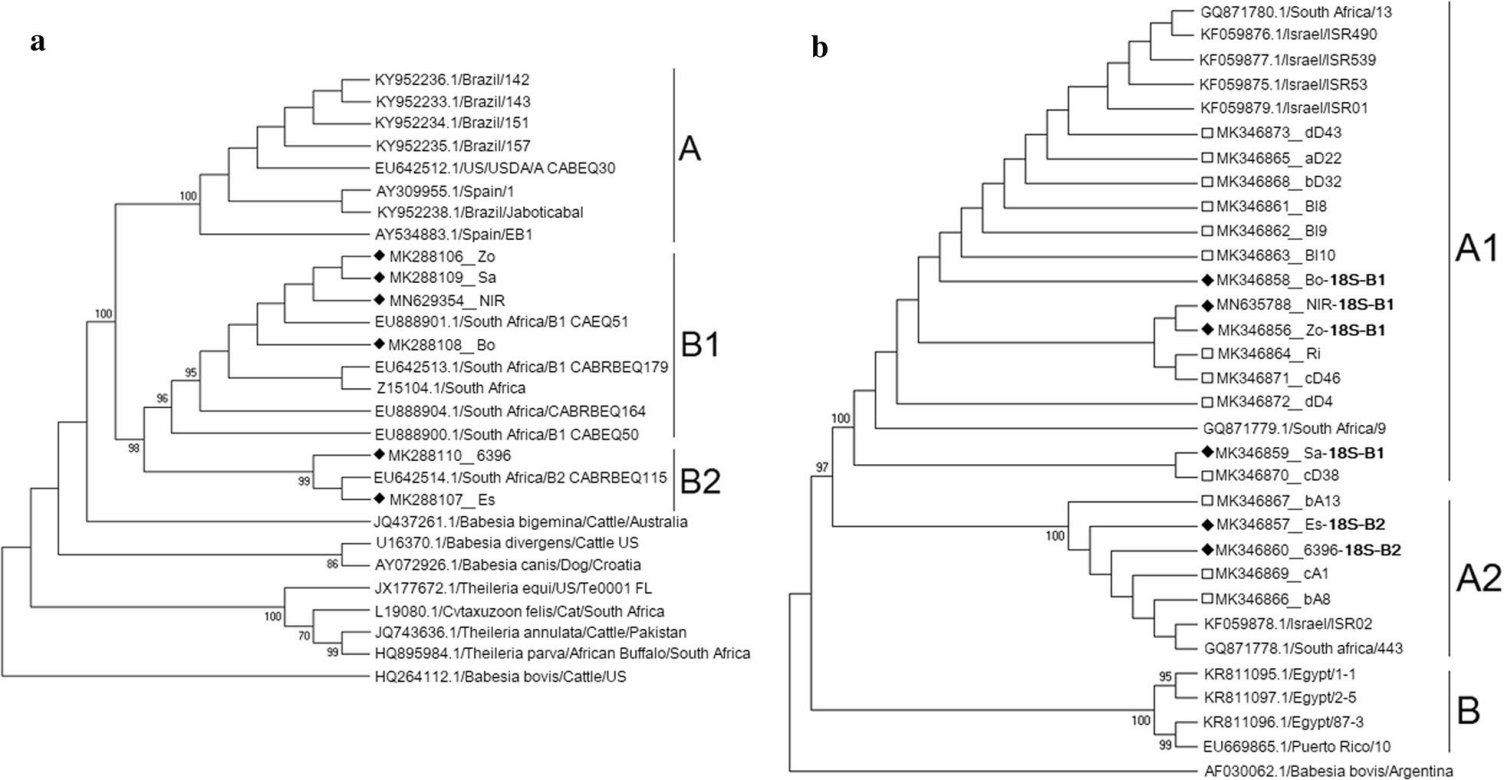
Phylogenetic analysis of *B. caballi* isolated from clinically infected horses (diamonds) and subclinically infected horses (open squares) (sample names as detailed in Additional file 1: Table S1). **a** Analysis of 1212 nucleotide positions of *B. caballi 18S* rRNA gene sequences from six clinical samples along with additional published sequences (GenBank ID/parasite/host/location). The phylogenetic tree was constructed based on the Tamura-Nei model with gamma distribution (+ G) and invariable sites (+ I). The percentage of trees in which the associated samples clustered together is shown next to the branches when it was above 70%. The analysis was constructed in MEGA7. **b** Analysis of 251 nucleotide positions of *B. caballi rap-1* gene sequences from six clinical and 13 subclinical samples, along with additional sequences from GenBank. The phylogenetic tree was based on the Kimura 2-parameter model with consideration on invariable sites (+ I). Both phylogenetic trees were constructed using maximum likelihood method and 1000 bootstrap replicates. The percentage of trees in which the associated samples clustered together is shown next to the branches when it was above 70%. The analysis was constructed in MEGA7

Of the two *B. caballi 18S* rRNA genotypes (A and B, with genotype B subdivided into subgroups B1 and B2) [6], the six clinical samples were classified as genotype B: four samples as genotype B1 and two samples as genotype B2 (Fig. 3a).

### Classification of *T. equi* based on *ema-1* and *ema-2* genes

Nine *T. equi ema-1* PCR products were successfully amplified and sequenced from four clinical and five subclinical horses (GenBank: MK415929-MK415937). Out of the three *T. equi ema-1* genotypes (A-C [14]), all samples were classified as genotype A (Fig. 2b).

*Theileria equi ema-2* PCR products were successfully amplified and sequenced from all six clinical and five subclinical horses (GenBank: MN624965-MN624979). The 11 sequences obtained in this study were analyzed with all available *T. equi ema-2* sequences on GenBank (20). Based on mean evolutionary distance (Additional file 4: Table S3), three genotypes were classified (A–C). The mean evolutionary distance within each genotype was under 0.004 base substitutions and the mean difference between groups was 0.011–0.059 base substitutions. All clinical and three subclinical isolates were classified as genotype A and two subclinical isolates were classified as genotype C (Fig. 2c).

Of the nine samples classified as *ema-2* genotype A, eight were also classified as *18S* rRNA genotype A and one as genotype C. Of the two samples classified as *ema-2* genotype C, one was *18S* rRNA genotype C and the other genotype D (Additional file 1: Table S1). The latter sequence was omitted from the phylogenetic analysis due to insufficient length.

### Classification of *B. caballi rap-1* gene

*Babesia caballi rap-1* gene was successfully amplified and sequenced from 19 horses, six showing clinical signs of disease and 13 subclinical carriers (GenBank: MK346858-MK346873, MN635788). A long fragment of over 1400 bp was obtained from all clinical and three subclinical cases; and a shorter fragment, between 250–1000 bp from the remaining samples.

Of the two *B. caballi rap-1* genotypes (A and B, with genotype A subdivided into subgroups A1 and A2) [12], four clinical and ten subclinical sequences obtained in this study were classified as genotype A1, while two clinical and three subclinical sequences were classified as genotype A2. All three subclinical isolates characterized as A2 originated from the same farm (Fig. 3b).

All clinical samples were classified based on both their *18S* rRNA and *rap-1* genes and showed association between the two. All four samples that were characterized as *18S* rRNA genotype B1 were also classified as *rap-1* genotype A1, while the remaining two samples were *18S* rRNA B2 and *rap-1* A2 (Additional file 1: Table S1).

## Discussion

Diagnosis of EP as the cause of clinical disease can be challenging in endemic areas, where the percentage of serologically and molecularly positive horses is high, and the detection of parasites does not necessarily imply on the cause of non-specific clinical signs [29]. Therefore, in clinical cases suspected as EP, quantitative evaluation of parasite load, using molecular tools, may assist in determining a threshold for cause of disease decision. Here we demonstrate that clinically infected horses with either parasite of EP have significantly higher parasite loads and lower PCV than subclinically infected horses. This is intuitive, as merozoite replication in erythrocytes causes hemolysis, the main clinical manifestation of EP. Thus, higher parasite loads may induce increased hemolysis that will be reflected in lower PCV. Parasite loads of both clinical and subclinical horses were generally lower in cases of *B. caballi* infection than in cases of *T. equi* infection. This may explain the milder clinical disease in *B. caballi* infections compared to *T. equi* infections, and to the possible natural clearance of *B. caballi* parasitemia without treatment, while *T. equi* carriage is usually life-long [1, 2].

To determine whether *T. equi* is the probable cause of disease in suspected clinical cases, we established a clear cut-off (*P* < 0.001) between clinical (0.12–3.8% PE) and subclinical (3 × 10^−4^–5 × 10^−3^% PE) cases. The parasitemia values in our study concur with published subclinical range (1.99–1000 parasites per µl blood [30], equivalent to 2.2 × 10^−5^ to 0.011% PE). In clinical cases, *T. equi* parasitemia ranges between 1–7% PE, and may reach up to 95% [1, 31]; however, we had clinical cases with parasitemia as low as 0.12% PE, which also presented with a low PCV. Three of six clinically *B. caballi* infected horses showed parasitemia below the documented range (0.1–10% PE) [1, 31]. Parasitemia in subclinical carriers of *B. caballi* ranged between 0.0001–0.0012% PE, which was significantly lower than the clinical cases (*P* < 0.001). To the best of our knowledge, no previous study quantified *B. caballi* parasitemia in subclinical horses. Although in this group, the difference between clinical and subclinical parasitemia was less distinct, it still resulted in a lower PCV, and allowed us to establish a cut-off value to identify *B. caballi* as the probable cause of clinical disease. Despite the limited number of cases included in the quantitative analysis, the highly significant results may serve as first indication that qPCR can be used as a diagnostic tool. Additional data should be collected to validate this method for clinical use.

All six clinical cases which originated from different farms and geographical locations were classified as *T. equi 18S* rRNA genotype A. The A genotype is not the most prevalent in Israel (30% of subclinical horses in this study, 33% in a previous study), and is rarely found in highly endemic farms [3]. Thus, although the number of cases was limited, genotype A may be associated with clinical disease. Genotype A was previously isolated from horses in both endemic and non-endemic countries [3, 6, 28, 32], it was isolated in two outbreaks in the USA [33], and was found to be associated with clinical and seropositive cases in Italy [19]. Interestingly, genotype A was the predominant genotype isolated from ticks collected from horses in Israel, including five different tick species of three genera. Some of these ticks were collected in farms in which this genotype was not isolated from horses [34, 35]. Interestingly, we found an association between *T. equi 18S* rRNA genotype and farm management (unpublished results). It is possible that this genotype is more adapted to the tick vector environment but encounters an active barrier during transmission to the host. Genotype A may also be less adapted to the host and may thus be more likely to result in clinical disease, whilst genotypes B, C and D are more likely to result in subclinical infection [19]. With the recent concerns regarding the classification of *Theileria* species according to the *18S* rRNA gene [7, 8], it is possible that this “A genotype” is the cause of the “classic” equine theileriosis, while other genotypes may represent closely related, less pathogenic, species or subspecies. More comprehensive genetic investigation of different genotypes is required to support this hypothesis.

In an attempt to partially address these issues, we classified *T. equi* according to three different genes: *18S* rRNA, *ema-1* and *ema-2*, as the last two loci had a sufficient number of published sequences for comparative analysis. However, we could not amplify *ema-1* and *ema-2* from all samples, probably due to polymorphism in the primer sites or the sensitivity of the PCR assay, and most of the successfully sequenced amplicons were from isolates of *18S* rRNA genotype A, as previously reported [19]. The overrepresented *18S* rRNA genotype A may be the result of the higher parasitemia in the clinically infected horses, enabling better detection by PCR. Nevertheless, using qPCR, *ema-1* gene was detected in all samples, strengthening parasite identification.

The *18S* rRNA classification in subclinical horses resulted in the detection of genotypes D (57.5%), A (30%) and C (12.5%) as previously described in our area [3], thus strengthening the statistical power of the data with a larger sample size. Sequence analysis of both e*ma-1* and *ema-2* did not reveal much polymorphism in these loci within a geographical area, as has been previously demonstrated [3, 14, 16–19]. Only 16 *ema-2* sequences were available for classification, mostly from India and the USA along with four sequences from Nigeria [36]. Although this gene had low variability, we identified three distinct genotypes, which also differ in their amino acid sequences. This variability may be important if it affects immune response and may also lower the sensitivity of the *ema-2* based ELISA assays [18].

Genetic classification of *B. caballi* is limited, with two *18S* rRNA genotypes identified in South Africa [6]. We were unable to amplify this gene from the subclinical horses, since all were co-infected with *T. equi*, and the primers are not species specific. Therefore, we used the *rap-1* gene which is specific to *B. caballi* and is fairly conserved, although with some heterogenicity between American and Asian-African strains [12–14, 19]. Two *18S* rRNA sub-genotypes were identified in clinical cases, which correlated with the *rap-1* sub-genotypes of the same samples. Comparison of the *rap-1* gene between clinical and subclinical cases did not reveal differences in parasite genotypes in relation to clinical disease.

The main limitation of this study was the small sample size of clinical cases. The number of clinical cases was limited mostly due to the relatively low occurrence of clinical cases in Israel. In addition, the relatively low prevalence of *B. caballi* in combination with high co-infection rates with *T. equi* prevented the establishment of a single-positive control group for this parasite and interfered with the ability to obtain good quality sequences of its *18S* rRNA gene. Lastly, in cases of *T. equi* infection, variations in the sensitivity of the PCR assays of the different loci did not allow the comprehensive analysis of all three genes from all samples. Nevertheless, the current groundwork brings unique quantitative data to be further supported by future studies.

## Conclusions

This study provides preliminary results supporting the use of quantitative molecular tools which assess parasite loads to help clinicians decide whether EP is the cause of the presenting clinical signs. In addition, *T. equi* genotype A (based on *18S* rRNA classification) was associated with clinical disease, while no such association was found for *B. caballi.* Future studies on parasites classification should be established in order to distinguish between closely related organisms or genotypes which may differ in their pathogenicity.

## Supplementary information

**Additional file 1: Table S1.** Detailed description of the study population, their clinical status and associated parasites of *B. caballi* and *T. equi.*

**Additional file 2: Table S2.** The PCR primers and probes used in this study.

**Additional file 3: Figure S1.** The geographical distribution of the collection sites of all clinical and subclinical equine cases of *T. equi* and *B. caballi* infection.

**Additional file 4: Table S3.** Estimates of the evolutionary divergence within and between *T. equi ema-2* genotypes.

## Data Availability

The datasets supporting the conclusions of this article are included within the article and its additional files.

## Abbreviations

ema: equi merozoite antigen
EP: equine piroplasmosis
gcn: gene copy number
PCV: packed cell volume
PE: parasitized erythrocytes
TS: total solids
rap: rhoptry associated protein
