# Supplementary material for: Parasite load and genotype are associated with clinical outcome of piroplasm-infected equines in Israel
Source: Parasit Vectors. 2020 May 20;13:267. doi: 10.1186/s13071-020-04133-y (PMC7240905; doi:10.1186/s13071-020-04133-y)
Supplement: Supplementary file 1 — Additional file 1: Table S1. Detailed description of the study population, their clinical status and associated parasites of B. caballi and T. equi. [file 13071_2020_4133_MOESM1_ESM.docx]

**Additional file 1: Table S1**

Detailed description of the study population. The characteristics of the horses and associated parasites of *B. caballi* and *T. equi* clinically (Y) and subclinically (N) infected horses. The sex, breed, age, geographical area and packed cell volume (PCV) are specified for each horse, along the molecular classification of isolated parasites according to the *18S rRNA*, *rap-1* (*B. caballi*)*, ema-1* and *ema-2* (*T. equi*(. S-stallion, M-mare, G-gelding; Ar-Arabian, TWH–Tennessee Walking Horse, QH-Quarter Horse, WB-Warmblood, An-Andalusian; GH-Golan Heights.

| **Organism** | **Clinical signs** | **ID** | **Sex** | **Breed** | **Age** | **Area** | **PCV** | **18S rRNA** | ***rap-1*** | ***ema-1*** | ***ema-2*** |
| --- | --- | --- | --- | --- | --- | --- | --- | --- | --- | --- | --- |
| *B. caballi* | Y | Zo | G | QH | 4 | Center |  | B1 | A1 |  |  |
| *B. caballi* | Y | 6393 | M | WB | 16 | Center | 27 | B2 | A2 |  |  |
| *B. caballi* | Y | Sa1 | M | QH |  | North |  | B1 | A1 |  |  |
| *B. caballi* | Y | Bo1 | M | QH | 5 | North | 17 | B1 | A1 |  |  |
| *B. caballi* | Y | Es | G | Mix | 2 | GH | 19 | B2 | A2 |  |  |
| *B. caballi* | Y | Nir | G | TWH | 4 | South | 16 | B1 | A1 |  |  |
| *B. caballi* | N | Ri | S | An | 19 | South | 29 | mixed | A1 |  |  |
| *B. caballi* | N | BI8 | M | Mix |  | Center |  | mixed | A1 |  |  |
| *B. caballi* | N | BI9 | M | Mix |  | Center |  | mixed | A1 |  |  |
| *B. caballi* | N | BI10 | G | Mix |  | Center |  | mixed | A1 |  |  |
| *B. caballi* | N | aD22 | G | Mix | 5 | North | 32 | mixed | A1 |  |  |
| *B. caballi* | N | bA8 | G | Mix | 3 | North | 29 | mixed | A2 |  |  |
| *B. caballi* | N | bA13 | G | Mix | 2 | North | 29 | mixed | A2 |  |  |
| *B. caballi* | N | bD32 | G | Mix | 8 | North | 31 | mixed | A1 |  |  |
| *B. caballi* | N | cA1 | G | Mix | 17 | North | 38 | mixed | A2 |  |  |
| *B. caballi* | N | cD35 | G | Mix | 8 | North | 33 | mixed | A1 |  |  |
| *B. caballi* | N | cD46 | G | Mix | 2 | North | 32 | mixed | A1 |  |  |
| *B. caballi* | N | dD4 | G | Mix | 2 | North | 27 | mixed | A1 |  |  |
| *B. caballi* | N | dD43 | G | Mix | 3 | North | 27 | mixed | A1 |  |  |
| *T. equi* | Y | Sma | G | QH | 10 | North | 17 | A |  | A | A |
| *T. equi* | Y | Gau | G | Ar | 15 | North | 20 | A |  | A | A |
| *T. equi* | Y | 52b | M | TWH | 17 | Center | 18 | A |  |  | A |
| *T. equi* | Y | Mish | M | QH | 17 | Center | 18 | A |  | A | A |
| *T. equi* | Y | BS | M | QH | 14 | North | 22 | A |  | A | A |
| *T. equi* | Y | BP | M | QH | 0 | North | 22 | A |  |  | A |
| *T. equi* | N | Kar | G | Mix |  | GH |  | A |  | A | A |
| *T. equi* | N | dA2 | G | Mix | 20 | North | 32 | D |  |  |  |
| *T. equi* | N | dA8 | G | Mix | 3 | North | 31 | D |  |  |  |
| *T. equi* | N | dA11 | G | Mix | 4 | North | 28 | C |  |  | C |
| *T. equi* | N | dA12 | G | Mix | 2 | North | 30 | C |  |  |  |
| *T. equi* | N | dD35 | G | Mix | 8 | North | 30 | A |  |  |  |
| *T. equi* | N | dD38 | G | Mix | 8 | North | 34 | D |  |  |  |
| *T. equi* | N | dE2 | M | Po | 15 | Center | 47 | A |  |  |  |
| *T. equi* | N | dE19 | M | Mix | 15 | Center | 34 | A |  |  |  |
| *T. equi* | N | dE37 | S | Po | 22 | Center | 35 | A |  |  |  |
| *T. equi* | N | dF8 | G | Mix | 10 | Center | 34 | A |  |  |  |
| *T. equi* | N | dF12 | M | Mix | 9 | Center | 37 | A |  |  |  |
| *T. equi* | N | dF13 | G | Mix | 17 | Center | 35 | D |  |  |  |
| *T. equi* | N | dF14 | M | Mix | 12 | Center | 34 | D |  |  |  |
| *T. equi* | N | dF16 | G | Mix | 12 | Center | 35 | A |  |  |  |
| *T. equi* | N | dI3 | G | QH | 6 | North | 38 | D |  |  |  |
| *T. equi* | N | dI9 | G | Mix | 14 | North | 36 | D |  |  |  |
| *T. equi* | N | dI12 | G | QH | 7 | North | 40 | A |  |  |  |
| *T. equi* | N | dI30 | M | QH | 15 | North | 33 | A |  | A |  |
| *T. equi* | N | dK1 | G | Mix | 21 | North | 35 | D |  |  |  |
| *T. equi* | N | dK4 | G | Mix | 10 | North | 38 | A |  |  | A |
| *T. equi* | N | dK10 | G | Mix | 4 | North | 26 | D |  |  | C |
| *T. equi* | N | dK13 | M | Mix | 5 | North | 36 | D |  |  |  |
| *T. equi* | N | dL4 | G | Mix | 9 | GH | 33 | D |  |  |  |
| *T. equi* | N | dL5 | M | Mix | 12 | GH | 30 | D |  |  |  |
| *T. equi* | N | dL6 | M | Mix | 5 | GH | 33 | D |  |  |  |
| *T. equi* | N | dL8 | G | Mix | 5 | GH | 33 | D |  |  |  |
| *T. equi* | N | dL9 | M | Mix | 6 | GH | 36 | D |  |  |  |
| *T. equi* | N | dM3 | M | Mix | 7 | GH | 37 | D |  |  |  |
| *T. equi* | N | dM10 | M | Mix | 8 | GH | 34 | C |  |  |  |
| *T. equi* | N | dM15 | G | Mix | 10 | GH | 32 | D |  |  |  |
| *T. equi* | N | dM30 | M | Mix | 4 | GH | 33 | C |  |  |  |
| *T. equi* | N | dR3 | M | Mix | 10 | North | 37 | D |  |  |  |
| *T. equi* | N | dR9 | G | QH | 9 | North | 32 | A |  | A |  |
| *T. equi* | N | dT1 | G | Mix | 4 | GH | 32 | D |  |  |  |
| *T. equi* | N | bT3 | G | Mix | 3 | GH | 28 | D |  |  |  |
| *T. equi* | N | dT4 | M | Mix | 12 | GH | 33 | D |  |  |  |
| *T. equi* | N | dT5 | M | Mix | 3 | GH | 33 | C |  |  | A |
| *T. equi* | N | dT7 | M | Mix | 6 | GH | 33 | D |  |  |  |
| *T. equi* | N | dT8 | G | Mix | 6 | GH | 32 | D |  |  |  |
| *T. equi* | N | dD24 | G | Mix | 5 | North | 35 |  |  |  | A |
