# Supplementary material for: Parasite load and genotype are associated with clinical outcome of piroplasm-infected equines in Israel
Source: Parasit Vectors. 2020 May 20;13:267. doi: 10.1186/s13071-020-04133-y (PMC7240905; doi:10.1186/s13071-020-04133-y)
Supplement: Supplementary file 2 — Additional file 2: Table S2. The PCR primers and probes used in this study. [file 13071_2020_4133_MOESM2_ESM.docx]

**Additional file 2: Table S2**

The PCR primers and probes used in this study.

| **Primer** | **Sequence 5'-3'** | **Target gene** | **Amplicon size (bp)** | **Reference** |
| --- | --- | --- | --- | --- |
| **Bec-UF2** | TCGAAGACGATCAGATACCGTCG | *T. equi/ B. caballi* 18S rRNA | 400 | 20 |
| **Equi-R** | TGCCTTAAACTTCCTTGCGAT |  |  |  |
| **Bc9_RAP2F** | ACTAGCGACCCCAACGCTACTGAC | *B. caballi Rap-1* | 400 | 11 |
| **Bc9_RAP2R** | TTGGAGCATGAAGTCCTTCAGC |  |  |  |
| **RT_EMAF** | CCGGCAAGAAGCACAYCTT | *T. equi ema-1* | 59 | 14 |
| **RT_EMAR** | TGCCATCGACGAYCTTGAG |  |  |  |
| **RT_EMA probe** | 6-FAM-TCCAGGCAAGCGC-MGB |  |  |  |
| **Bc_18SF402** | GTAATTGGAATGATGGCGACTTAA | *B. caballi* 18S rRNA | 95 | 22 |
| **Bc_18SR496** | CGCTATTGGAGCTGGAATTACC |  |  |  |
| **Bc_18SP** | 6-FAM-CCTCGCCAGAGTAA-MGB |  |  |  |
| **NBabesia1F** | AAGCCATGCATGTCTAAGTATAAGCTTTT | *T. equi* 18S rRNA | 1600 | 25 |
| **18SRev-TB** | GAATAATTCACCGGATCACTCG |  |  | 24 |
| **BT18S2F** | GGGTTCGATTCCGGAGAGGG | *T. equi* 18S rRNA | 800 | 25 |
| **BT18S2R** | CCCGTGTTGAGTCAAATTAAGCCG |  |  | 24 |
| **BT18S3F** | GGGCATTCGTATTTAACTGTCAGAGG | *T. equi* 18S rRNA | 800 | 25 |
| **BT18S3R** | CCTCTGACAGTTAAATACGAATGCCC |  |  | 5 |
| **EMA-1F** | GCATCCATTGCCATTTCGAG | *T. equi ema-1* | 750 | 20 |
| **EMA-1R** | TGCGCCATAGACGGAGAAGC |  |  |  |
| **EMA-2F** | AATGTTGAGCAAGTCCTTCG | *T. equi ema-2* | 800 | Kumar et al., 2018* |
| **EMA-2R** | TTAGTAGAACAAAGCAACGGC |  |  |  |
| **Bc9_RAPF** | AGCAGTGCTGTATATGTCTGTGTC | *B. caballi rap-1* | 1500 | 11 |
| **Bc9_RAPR** | GCTGATGCGATGTGTGTCGTAGG |  |  |  |

* according to GenBank submissions (KC347578)
