# Supplementary material for: Parasite load and genotype are associated with clinical outcome of piroplasm-infected equines in Israel
Source: Parasit Vectors. 2020 May 20;13:267. doi: 10.1186/s13071-020-04133-y (PMC7240905; doi:10.1186/s13071-020-04133-y)

**Additional file 3: Figure S1**

The geographic distribution of the collection sites of all clinical and subclinical equine cases of *T. equi* (TE, red) and *B. caballi* (BC, blue) infection. The number of subclinical cases in each site is represented by the size of the symbol (circles for TE, squares for BC). Only one clinical case was obtained from each site (triangles for TE, diamonds for BC).


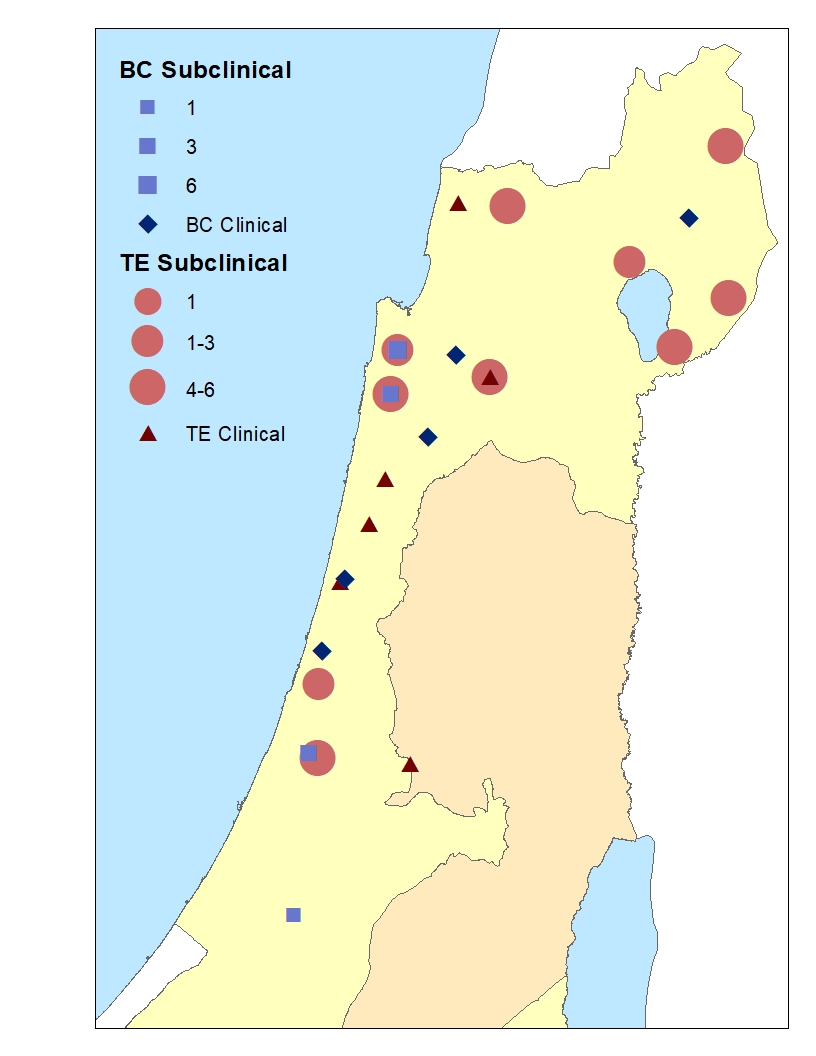

Supplement: Supplementary file 3 — Additional file 3: Figure S1. The geographical distribution of the collection sites of all clinical and subclinical equine cases of T. equi and B. caballi infection. [file 13071_2020_4133_MOESM3_ESM.docx]
