# Supplementary material for: Parasite load and genotype are associated with clinical outcome of piroplasm-infected equines in Israel
Source: Parasit Vectors. 2020 May 20;13:267. doi: 10.1186/s13071-020-04133-y (PMC7240905; doi:10.1186/s13071-020-04133-y)
Supplement: Supplementary file 4 — Additional file 4: Table S3. Estimates of the evolutionary divergence within and between T. equi ema-2 genotypes. [file 13071_2020_4133_MOESM4_ESM.docx]

**Additional file 4: Table S3**

Estimates of the evolutionary divergence within and between *T. equi ema-2* genotypes. The average number of base substitutions per site is shown. A total of 782 nucleotide positions of 29 nucleotide sequences were analyzed. The analyses were conducted using Kimura-2 parameter+G medel in MEGA7.

|  | **Within group** | **Between groups** | |
| --- | --- | --- | --- |
| **Genotype** |  | **A** | **B** |
| **A** | 0.000 |  |  |
| **B** | 0.004 | 0.011 |  |
| **C** | 0.001 | 0.059 | 0.055 |
